# Screening for Cardiac Disease-Induced Post-Traumatic Stress Disorder among the Partners of Cardiac Disease Patients: Unheard Murmurs of Malaysian Heart

**DOI:** 10.21315/mjms2022.29.2.16

**Published:** 2022-04-21

**Authors:** Ramasamy CHIDAMBARAM

**Affiliations:** Department of Prosthodontics, Faculty of Dentistry, SEGI University, Selangor, Malaysia

Dear Editor,

The gradual increase in stress following a trauma not limited to serious injury, war, accident, chronic illness, domestic violence, sexual abuse and personal threat often transforms into post-traumatic stress disorder (PTSD) in the victims. However, the family members providing care to the victims might also be at risk of developing PTSD. One such unrecognised threat, referred to as the cardiac disease-induced-PTSD (CDI-PTSD), occurs in the partners of the cardiac disease (CD) patients. Although high comorbidities accompany PTSD, there is a reason for targeting the CD population, particularly the partners of the CD patients. The changes in the lifestyle of people and the associated stress have mutually contributed to the increasing incidence of CD worldwide, including Malaysia. Unfortunately, CD remains the leading cause of death in Malaysia consecutively for the 14th year and the number of deaths is increasing gradually since 2010 (9,371), with the numbers doubled to 18,267 (1). The shocking figures have rendered it imperative to find ways to limit the number of new admissions with CD.

On all grounds, partners are the most proximate ones to individuals. As both partners age together, one may require special attention while the other provides the required care and support. The future is therefore predictable in such circumstances. In terms of cardiac event, the mental trauma experienced by the partner of the CD patient is worse as the partner remains conscious throughout the event and treatment process, while the patient is unconscious. Pioneers such as the American Psychiatric Association claim that witnessing a cardiac event equally predisposes the witness to PTSD (2) and this one factor differentiates CD from all the other traumatic events, encouraging the research focused on the potential traumatised partners of CD patients.

A sedentary lifestyle is a leading factor contributing to cardiovascular risks in Malaysia. The individuals who mostly experience a CD event are midgets with their respective spouses as primary caregivers (3). Findings of the American Heart Association strongly suggest that the partners of CD patients have an increased risk of disturbed physical and psychological health (4). Furthermore, the assisting partner may not even realise that they are becoming the caregiver and are being exposed to mental trauma. The psychological outcomes in caregivers are directly related to the patient’s health, and the resulting distress may ultimately affect the quality of care provided to the patient and put them at a higher medical risk. Therefore, it is reasonable to state that the partners of CD patients are at a high risk of developing CDI-PTSD.

Innovative thinking during the screening of the partners of CD patients is crucial to identify the subjects at a high risk of PTSD. Identification of PTSD, which is a risk factor for CD, would provide an opportunity to reduce distress and limit the new admissions with CD. The recently validated Malay post-traumatic stress disorder checklist (MPCL-5) could serve the purpose (5) and we believe that our devoted doctors would consider it important to screen the partners of the CD patients for CDI-PTSD.
